# A Mini-Review on Cell Cycle Regulation of Coronavirus Infection

**DOI:** 10.3389/fvets.2020.586826

**Published:** 2020-11-05

**Authors:** Mingjun Su, Yaping Chen, Shanshan Qi, Da Shi, Li Feng, Dongbo Sun

**Affiliations:** ^1^Laboratory for the Prevention and Control of Swine Infectious Diseases, College of Animal Science and Veterinary Medicine, Heilongjiang Bayi Agricultural University, Daqing, China; ^2^State Key Laboratory of Veterinary Biotechnology, Harbin Veterinary Research Institute, Chinese Academy of Agricultural Sciences, Harbin, China

**Keywords:** coronavirus, cell cycle arrest, cyclin-CDK complex, p53, nucleocapsid protein

## Abstract

Coronaviruses are widespread in nature and infect humans, mammals and poultry. They cause harm to humans and animals. Virus-mediated cell cycle arrest is an essential strategy for viral survival and proliferation in the host cells. A clarification system of the mechanisms of virus-induced cell cycle arrest is highly desirable to promote the development of antiviral therapies. In this review, molecular mechanisms of coronavirus-induced cell cycle arrest were systematically summarized. Moreover, the common features of coronavirus-mediated cell cycle arrest were discussed. This review will provide a theoretical basis for further studies on the infection mechanisms and prevention of coronaviruses.

## Introduction

Coronaviruses (CoVs) are the largest positive-sense, single-stranded RNA viruses. CoVs belong to the Coronaviridae family of the Nidovirales order. CoVs are classified into four genera: Alphacoronavirus (α-CoV), Betacoronavirus (β-CoV), Gammacoronavirus (γ-CoV), and Deltacoronavirus (δ-CoV) (1). CoVs share a common basic genomic organization. The genome size is approximately 25000–30000 nt. CoV contains at least six open reading frames (ORFs) in the order: ORF1a, ORF1b, spike (S), envelope (E), membrane (M), and nucleocapsid (N), and certain CoVs encode a hemagglutinin esterase (HE). Each virus contains one to several accessory genes, the ORF3 of α-CoV porcine epidemic diarrhea virus (PEDV), the ORF3a, ORF3b, ORF6, ORF7a, ORF7b, ORF8a, ORF8b, and ORF9 of severe acute respiratory syndrome coronavirus (SARS-CoV), the ORF3a, ORF3b, ORF5a, ORF5b of γ-CoV infectious bronchitis virus (IBV), NS6 and NS7 of δ-CoV porcine deltacoronavirus (PDCoV). Several large-scale epidemics of human and animal CoVs have occurred over the last few decades, especially the SARS-CoV outbreak in 2003, the Middle East respiratory syndrome coronavirus (MERS-CoV) outbreak in 2012, and the current global pandemic of severe acute respiratory syndrome coronavirus 2 (SARS-CoV-2), which has threatened global public health security. Therefore, CoVs have become a topic of research interest in the field of virology.

The interaction between the virus and the host is a symbiotic relationship. As obligate parasites, viruses entirely depend on the cellular pathways of the host to obtain resources necessary for replication. Eukaryotic cell cycle progression is a highly ordered and tightly regulated process that performs a crucial role in maintaining cell metabolism and homeostasis. When cells are stressed or injured, the host cells initiate repair mechanisms by regulating cell cycle progression, which is a primary response to external stimulation. Several viruses often interfere with the normal cell cycle progression to obtain sufficient resources for viral replication by inducing host cell cycle arrest in an active metabolic state, such as the DNA synthesis phase (2–4). Studies have confirmed that CoVs such as transmissible gastroenteritis virus (TGEV), PEDV, murine hepatitis virus (MHV), SARS-CoV, and IBV induce cell cycle arrest to facilitate viral replication (5–19). Although studies on CoV-regulated cell cycle have had a high research output, there has been no systematic overview of the studies. In this review, we aimed to summarize molecular mechanisms that are used by CoVs to manipulate the cell cycle and to provide insights into the common features of CoV-mediated cell cycle arrest.

### Cell Cycle and Regulation

A eukaryotic cell cycle comprises four different phases: Gap 1 (G1), Synthesis (S), Gap 2 (G2), and Mitosis (M) (20, 21). During the G1 phase, the cell primarily synthesizes proteins, saccharides and RNA, which are necessary for DNA replication in preparation for entry into the S phase (DNA synthesis phase). After DNA replication is completed, the cell enters the G2 phase and prepares for mitosis, cell division. Cyclin-dependent kinases (CDKs) and cyclins are the major regulators of cell cycle progression (20, 21). In mammalian cells, different cyclin-CDK complexes are involved in the regulation of different cell cycle transitions (20, 21). During the G1 phase, cyclin D/CDK4 (or CDK6) complexes phosphorylate the downstream factor, retinoblastoma (Rb), which in turn releases the transcription factor E2F promoting transcription of cyclin E, cyclin A, and CDK1 genes (22, 23). Transition from G1 to S phase is regulated by the cyclin A-CDK2 and cyclin E-CDK2 complexes (24). During the G2 phase, the cyclin A (or cyclin B)-CDK1 complexes phosphorylate histone H1, which initiates the cells into the M phase (25, 26). In addition, the cell cycle is negatively regulated by CDK inhibitors (CKIs) (20, 21). CKIs such as p21, can either bind to isolated CDKs or to the cyclin-CDK complex to prevent activation of CDKs. Furthermore, the tumor suppressor p53 that indirectly regulates more than 250 cell cycle-associated genes, performs an essential role in the regulation of the cell cycle (27, 28). Activation and induction of p53 in response to DNA damages or other stresses, and upregulation of p21 expression can inhibit CDK activity, thereby inducing cell cycle arrest or apoptosis.

### Cell Cycle Regulation of CoVs Infection

CoVs are pathogens with a wide range of hosts that critically endanger human health and development of animal husbandry. Notably, the prevention and control of emerging and re-emerging CoVs such as SARS-CoV-2 and PEDV, has been a great challenge globally. Therefore, in addition to the development of diagnostic reagents, antiviral drugs, and vaccines, it is imperative to have a deeper understanding of the interaction mechanisms between CoVs and host cells.

**Figure 1:**
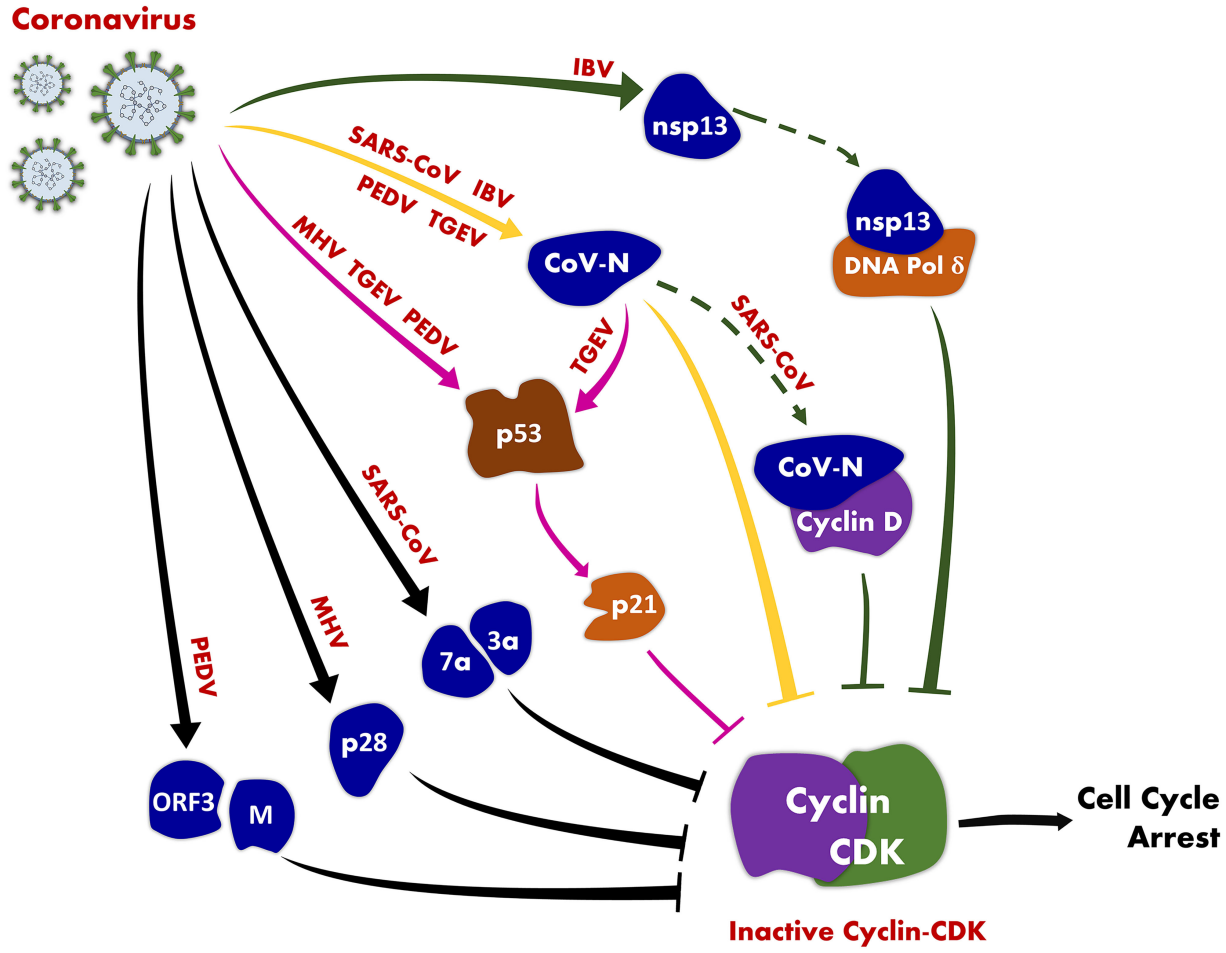
An illustration of coronavirus-induced cell cycle arrest. CoV induces host cell cycle arrest by inhibiting the activity of cyclin-CDK complexes through the following pathways: CoV (MHV, TGEV, and PEDV) inhibits the activity of cyclin-CDK complexes through the p53 signaling pathway (pink solid lines); CoV N protein (SARS-CoV, TGEV, and PEDV) suppresses the activity of cyclin-CDK complexes (yellow solid lines); CoV (SARS-CoV, IBV) inhibits the activity of cyclin-CDK complexes by directly interacting with host cell cycle-associated proteins (solid and dotted green lines).

CoV-regulated cell cycle has become one of the research hot spots in CoVs research in recent years. Viruses have evolved multiple mechanisms that induce cell cycle arrest to generate resources and cellular conditions favorable for viral replication to enhance replication efficiency. Numerous CoVs have evolved diverse strategies to manipulate host cell cycle for their own replication (Table 1, Figure 1). The various types of CoVs not only exhibit distinct characteristics of cell cycle regulation, but also exhibit potentially common regulatory mechanisms.

**Table 1:** The information of cell cycle arrest by coronaviruses.

**Coronaviruses**	**Cell cycle arrest by coronaviruses**
Transmissible gastroenteritis virus, TGEV	S and G2/M phase (18, 19)
Porcine hemagglutinating encephalomyelitis virus, PHEV	NA
Porcine epidemic diarrhea virus, PEDV	G0/G1 phase (12, 13); S phase (5, 14, 15)
Porcine respiratory coronavirus, PRCV	NA
Porcine deltacoronavirus, PDCoV	NA
Swine acute diarrhea syndrome coronavirus, SADS-CoV	NA
Canine coronavirus, CCoV	NA
Canine respiratory coronavirus, CRCV	NA
Pantropic canine coronavirus, PCCoV	NA
Feline infectious peritonitis virus, FIPV	NA
Feline enteric coronavirus, FECV	NA
Infectious bronchitis virus, IBV	S and G2/M phase (16, 17)
Turkey coronavirus, TCoV	NA
Murine hepatitis virus, MHV	G0/G1 phase (10, 11)
Sialodacryoadenitis virus, SDAV	NA
Bovine coronavirus, BCoV	NA
Equine coronavirus, ECoV	NA
Human coronavirus 229E, HCoV-229E	NA
Human coronavirus NL63, HCoV-NL63	NA
Human coronavirus HKU1, HCoV-HKU1	NA
Severe acute respiratory syndrome coronavirus, SARS-CoV	G0/G1 phase (6–8); S phase (9)
Middle East respiratory syndrome coronavirus, MERS-CoV	NA
Severe acute respiratory syndrome coronavirus 2, SARS-CoV-2	NA

### CoVs Manipulate the Cell Cycle by Regulating Cyclin-CDK Complex

Cyclin-CDK complexes are the core components of the cell cycle regulatory network. Similar to other viruses, CoVs affect the activity of cyclin-CDK complexes, which is a common strategy of manipulating cell cycle progression. SARS-CoV accessory proteins, 3a and 7b inhibit Rb phosphorylation by limiting the expression of cyclin D3, which in turn blocks the G0/G1 phase of the cell cycle in HEK 239 cells (7, 8). The N protein of SARS-CoV inhibits the activity of cyclin-CDK complex and blocks progression of the S phase in Huh7 cells (9). MHV-infected cells cause a reduction in the amounts of CDKs (CDK4 and CDK6) and cyclins (cyclin D1, D2, D3, and E) in 17Cl-1 cells, and insufficient hyperphosphorylation of pRb, resulting in inhibition of the cell cycle in the G0/G1 phase (10, 11). PEDV infection can induce cell cycle arrest in the G0/G1 phase in Vero cells, by up-regulating p21, cdc2, CDK2, CDKk4, cyclin A, cyclin B1 protein and down-regulating cyclin D1 and cyclin E1 protein (12, 13). However, other studies have revealed that PEDV N and M proteins prolong the S phase in IEC cells by down-regulating cyclin A (14, 15). IBV infection induces cell cycle arrest in the S and G2/M phases in Vero cells, by down-regulating cyclin D1 and cyclin D2, and the redistribution of subcellular location of cyclin D1 was observed (16, 17). TGEV N proteins induce accumulation of PK-15 cells in the S and G2/M phases by inhibiting expression of cyclin B1 and CDK2 (18).

### CoVs Manipulate Cell Cycle Through p53-Dependent Pathway

The tumor suppressor, p53 has a key role in modulating response to cellular stress (27, 28). Activated p53 regulates hundreds of genes involved in multiple biological processes, including DNA damage repair, cell cycle arrest, apoptosis and senescence. Furthermore, p53 is involved in the manipulation of virus-host cell cycle. Previous studies have reported that CoV infections induce cell cycle arrest via the p53-dependent pathway (11, 12, 18, 19). MHV, PEDV and TGEV induce p53 activation and upregulate the expression of p21, which binds to cyclin-CDK complexes and inhibits kinase activity resulting in host cell cycle arrest. In addition, a previous study revealed that p53 phosphorylation on Ser-15 was upregulated in both 3a and M proteins of SARS-CoV expressed in HEK 239 cells (7); SARS-CoV and HCoV-NL63 infections affect the degradation pathway of p53 (29, 30); IBV infection redistributes the subcellular location of p53 (31). Such studies indicate that p53 has a crucial role in CoV-mediated cell cycle arrest. However, a previous study indicated that cell cycle arrest induced by IBV infection in H1299 cells was p53-independent (16). Therefore, further studies are required to investigate if p53 is a common regulatory mechanism of the CoV-mediated cell cycle arrest.

### Cell Cycle Regulation by N Protein of CoVs

The N protein of CoVs is a multifunctional protein involved in multiple steps of viral replication (32). The N protein of CoV has been demonstrated to have a key role in regulating cell cycle progression, such as apoptosis, autophagy, and antagonizing innate immune responses (18, 33, 34). The N protein also modulates host cell cycle. Previous studies have reported that SARS-CoV, PEDV, and TGEV N proteins induce host cell cycle arrest in the S phase (9, 15, 18). SARS-CoV N protein inhibits the activity of cyclin-CDK complex by directly binding to cyclin D or cyclin-CDK2 complex, and by indirectly down-regulating protein levels of CDK2, cyclin E, and cyclin A (9). The nucleolus is a sub-nuclear compartment associated with numerous biological processes, including the cell cycle (35). Nuclear localization of the N protein is a common feature of CoVs, and the N protein interacts with nucleolar proteins such as nucleophosmin (NPM1) and fibrillarin (34, 36). In addition, Wurm et al. (35) reported that nucleolar localization of N proteins correlates with the disruption of host cell division caused by CoVs (37). A study by Cawood et al. (38) revealed that the different stages of the cell cycle can affect dynamic trafficking of N proteins in the nucleus and nucleolus (38). Moreover, Liu et al. (39) demonstrated that PEDV-induced cell cycle arrest in Vero cells depended on the nuclear localization signals (39).

### CoVs Manipulate the Cell Cycle by Directly Interacting With Host Cell Cycle-Associated Proteins

The direct interaction between viral and host proteins is one of the effective strategies used by virus to manipulate the host cell cycle (40, 41). CoVs can subvert the host cell cycle to facilitate viral replication through its interactions with host proteins. SARS-CoV N protein prolongs cell cycle of the S phase by directly interacting with cyclin D (9). Nsp13 proteins of IBV and SARS-CoV interact with the p125 subunit of DNA polymerase δ to induce DNA damage, consequently causing cell cycle arrest in the S phase (42). MHV nsp15 interacts with pRb, increases expression of genes that are normally repressed by pRb, and affects the cell cycle by blocking cell cycle progression at the S phase in NIH 3T3 cells (43). In addition, binding of PEDV N protein with NPM1 prevents proteolytic cleavage of NPM1 and enhances survival of Vero cells (34). Moreover, PEDV M protein interacts with the cell cycle-associated protein cell division cycle 42 (CDC42) (44).

## Prospects

The molecular mechanisms of CoV-mediated regulation of cell cycle have not been systematically investigated, especially the common features of CoV-mediated cell cycle arrest. Further studies should be conducted in the future to explore the mechanisms according to the characteristics of CoVs and regulation of the cell cycle induced by each CoV. The N protein of CoVs and p53 of host proteins that are the key factors of coronavirus-mediated cell cycle regulation in this review form potentially valuable foundations for future studies on the common mechanisms of CoV-mediated cell cycle arrest.

Virus infection is a complex, multistage, and highly dynamic process. Virus dynamic regulates progression of the host cell cycle at various phases of infection based on the replication requirements. For example, certain viruses can induce cell-cycle arrest during the initial stages of a viral infection to utilize a host's resources, subsequently resulting in apoptosis of host cells for virion release (40). A few studies have been conducted on the dynamics of regulation of the host cell cycle process in CoVs, and the studies have focused on the effect of the virus on cell cycle at a specific stage of infection, and individual viral proteins on the cell cycle. This has resulted in the negligence of the impacts of different phases of infection and synergy between viral proteins and host cell cycle progression, resulting in limited understanding of the host cell cycle regulation during a CoV infection.

To date, numerous studies on the regulation of cell cycle by CoVs are based on grounded theory to explore how viruses regulate the cell cycle, and a few are based on applied research. Targeted blocking of key pathways by designing small-molecule drugs based on the pathways of viral regulation of the cell cycle is an effective strategy of inhibiting viral replication. CoVs can convert the host cell cycle into a microenvironment for virus proliferation through direct interaction with host cell cycle-associated proteins. Based on this, the interaction sites may be an ideal target for the small molecule drugs. In addition, based on the viral replication requirements, the microenvironment for viral replication can be enhanced by optimizing the cell culture media for viral vaccines, which can enhance the efficiency of vaccine production. However, this has not been successfully applied in the production of vaccines due to lack of systematic scientific theories.

## Author Contributions

DS: conceptualization. MS, SQ, DS, and LF: investigation. MS: writing. MS and YC: revision. All authors contributed to the article and approved the submitted version.

## Conflict of Interest

The authors declare that the research was conducted in the absence of any commercial or financial relationships that could be construed as a potential conflict of interest.
